# Sex difference in incidence of major depressive disorder: an analysis from the Global Burden of Disease Study 2019

**DOI:** 10.1186/s12991-023-00486-7

**Published:** 2023-12-12

**Authors:** Sangzi Li, Xuan Zhang, Yilu Cai, Leilei Zheng, Hu Pang, Lixia Lou

**Affiliations:** 1 School of Design and Fashion, Zhejiang University of Science and Technology, Hangzhou, China; 2 School of Marxism, Zhejiang University, Hangzhou, China; 3 Eye Center, The Second Affiliated Hospital, School of Medicine, Zhejiang University, Hangzhou, China; 4 Department of Psychiatry, The Second Affiliated Hospital, School of Medicine, Zhejiang University, Hangzhou, China

**Keywords:** Major depressive disorder, Sex difference, Incidence, Trend, Age, Socioeconomic development

## Abstract

**Background:**

Major depressive disorder (MDD) is a leading mental disorder causing severe impairment. This study was aimed to evaluate sex difference in global MDD incidence by year, age, and socioeconomic status, according to the Global Burden of Disease Study 2019 (GBD 2019).

**Methods:**

Global and national sex-specific incidence estimates of MDD, from 1990 to 2019, in different age groups, were extracted from the GBD 2019. Socioeconomic development index (SDI) as an indicator of national socioeconomic development was used. Absolute (female minus male) and relative (female to male ratio) sex difference in age-standardized incidence rates (ASRs), as well as risk ratios (RR and 95% confidence interval), were computed by year and age. Linear regression analyses were conducted to investigate socioeconomic-associated sex difference in incidence.

**Results:**

Absolute and relative sex difference in ASRs showed a slight declining trend during 1990 and 2019, with absolute difference decreasing from 1818.23 to 1602.58, and relative difference decreasing from 1.71 to 1.61. Worldwide, females had a higher risk of MDD than males in 1990 (RR: 1.706 (1.705–1.706)) and 2019 (RR: 1.602 (1.619–1.620)). The highest RRs were observed in the Region of the Americas. Sex difference in incidence rates increased rapidly with age for those under 20 years old. The highest RR (1.913 (1.910–1.915)) was observed in the age group of 10–14. Relative sex difference had a significant positive relationship with SDI (standardized β = 0.267, P < 0.001).

**Conclusions:**

Despite that slight improvement in sex difference in global MDD incidence has been achieved, sex difference still persists in the past decades, with females always having a higher incidence than males. Greater sex difference was found at younger ages and in more developed countries. The findings highlight the importance of making sex-specific health policy to reduce sex difference in MDD incidence.

## Introduction

Depression is a common illness that severely affects psychosocial function and lowers quality of life [1]. According to the Global Burden of Disease study 2019 (GBD 2019), depression accounted for the largest proportion (37.3%) of disability-adjusted life years caused by mental disorders in 2019, followed by anxiety (22.9%), and schizophrenia (12.2%) [2]. Major depression disorder (MDD) is an episodic mood disorder with shorter duration but more severe symptoms than dysthymia. Globally, approximately 185 million people suffer from MDD [3], which could cause difficulties in all aspects of life, including at home, work and school. Persistent depression is also related to negative clinical conditions (e.g., suicidal behavior) [4]. The Hypothalamus–Pituitary–Adrenal (HPA) axis abnormalities, mainly characterized by hyperactivity of the HPA axis, may exert an important modulatory influence on suicide risk, regardless of the presence or absence of psychiatric conditions [5]. Epidemiological research has shown substantial gender-related difference in prevalence for depression, with 170.4 per million in females versus 109.2 per million in males [2]. Our previous analysis of the GBD 2019 data found that the health burden measured by disability-adjusted life-years (DALYs) was much higher in females than that in males, with age-standardized DALY rates (per 100,000 population) being 564 versus 354 in 2019 [6]. A complex interaction of social, psychological, and biological factors could contribute to female vulnerability to MDD [7, 8].

Although psychological therapy and antidepressant medications are effective treatments for MDD [9], sex difference in MDD burden remains a major global public health problem that requires attention from policymakers and health planners around the world. Regional studies from USA, Canada and Ethiopia have indicated higher incidence of MDD in females than males across all age groups [10]. The global patterns of sex difference in MDD incidence are important for making sex-specific health policy to reduce MDD. Thus, the aim of this study was to evaluate sex difference in global MDD incidence by year, age, and socioeconomic status, using the most up-to-date data from the GBD 2019 [11]. It should be noted that new GBD 2021 estimates are going to be published in the next months. COVID-19 pandemic may have changed the burden of MDD, and no mention on it was provided in this study.

## Materials and methods

### Sex-specific incidence of major depressive disorder

The GBD 2019 estimated incidence of MDD, for males and females, 20 age groups, 204 countries and territories, from 1990 onwards. The GBD study applies the Bayesian meta-regression tool DisMod-MR 2.1to utilize all accessible information (including published literature, surveillance data, survey data, hospital and clinical data, and other types of data) on MDD occurrence ﻿that passes a set of inclusion criteria, to measure the incidence of MDD [12]. ﻿Statistical code used for GBD estimation is publicly available online [13]. Incidence rates are expressed as age-standardized based on the GBD reference population [14]. The GBD study applies the same technique for propagating uncertainty as used elsewhere [11].﻿ Final estimates are computed using the mean estimate across 1000 draws, and the 95% uncertainty intervals (UIs) are determined on the basis of the 25th and 975th ranked values across all 1000 draws. To allow for comparability, the definition of MDD in the GBD 2019 adheres to DSM-IV-TR or ICD-10 criteria [2]. The following data concerning MDD were extracted from the GBD Results Tool [3]: (i) global age-standardized incidence rates (ASRs) (per 100,000 population), for males and females, from 1990 to 2019; (ii) global and World Health Organization (WHO) regional incident cases and ASRs, for males and females, in 1990 and 2019; (iii) global incidence rates (per 100,000 population), for males and females, 20 age groups, in 2019; (iv) national ASRs, for males and females, 204 countries and territories, in 2019.

### Socioeconomic development index

The socioeconomic development index (SDI) is a composite indicator ﻿of a country’s lag-distributed income per capita, average years of schooling, and the fertility rate in females under the age of 25 years, comprehensively assessing socioeconomic development status [11]. ﻿The GBD study computed the composite SDI as the geometric mean of the three above mentioned covariates, using the Human Development Index methodology [15]. The cutoff values used to determine quintiles for analysis were then computed using country-level estimates of SDI for 2019. The values of SDI range from 0 to 1, with a higher value reflecting better socioeconomic development. 204 countries and territories were classified into five groups by SDI in 2019 [11], including 33 in low SDI group (0 < SDI < 0.45), 43 in low-middle SDI group (0.45 ≤ SDI < 0.61), 41 in middle SDI group (0.61 ≤ SDI < 0.69), 48 in high-middle SDI group (0.69 ≤ SDI < 0.81), and 39 in high SDI group (0.81 ≤ SDI < 1).

### Statistical analysis

Global absolute (female minus male) and relative (female to male ratio) sex difference in incidence, as well as risk ratios (RR) and 95% confidence intervals (CI), were computed by year and age. ASRs for males and females across 204 countries and territories were compared using Mann–Whitney U test [16], with multiple comparisons between different SDI groups. Linear regression analyses were performed to evaluate the association of absolute (female minus male) and relative (female to male ratio) sex difference in ASRs with SDI across 204 countries and territories. All statistical analyses were conducted using SPSS 23 (IBM, Chicago, USA). P values less than 0.05 were considered statistically significant.

## Results

### Sex difference in incidence by year

**Fig. 1:**
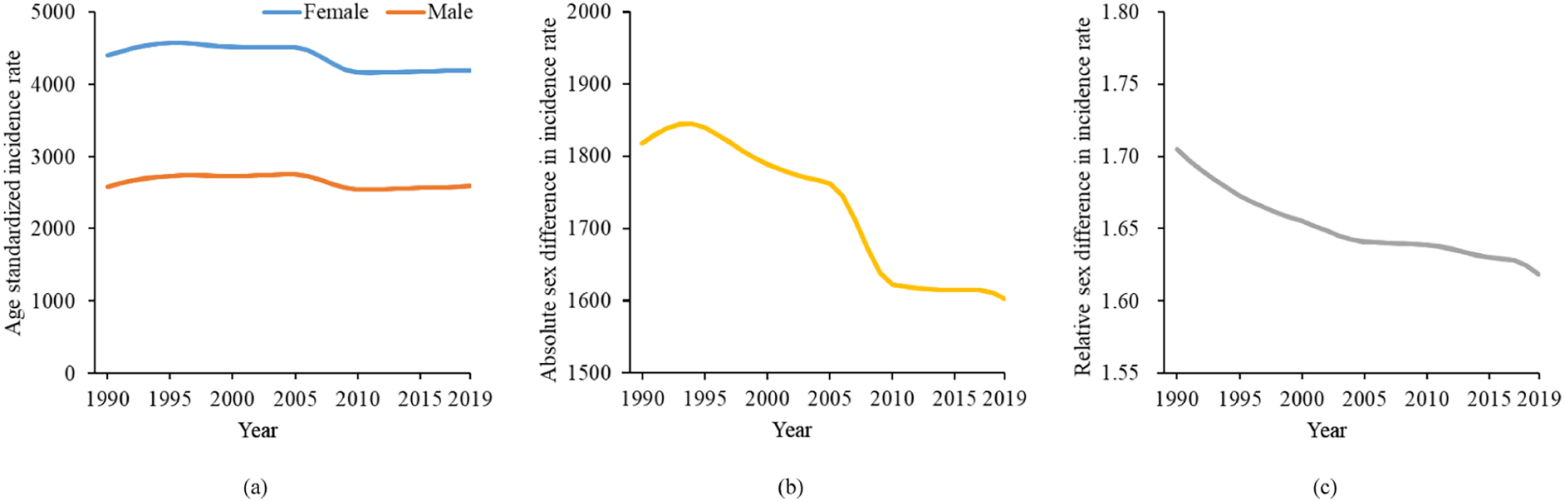
Sex difference in global incidence of major depressive disorder from 1990 to 2019, in terms of **a** age standardized incidence rates, **b** absolute (female minus male) sex difference in age standardized incidence rates, and **c** relative (female to male ratio) sex difference in age standardized incidence rates

From 1990 to 2019, ASRs of MDD in both sexes did not change so much at the global level, with ASRs being 2578.85 (95% UI: 2266.15–2925.51) in 1990 and 2591.15 (95% UI: 2269.86–2942.69) in 2019 for males, and being 4397.08 (95% UI: 3869.11–4976.14) in 1990 and 4193.73 (95% UI: 3672.83–4777.14) in 2019 for females (Fig. 1a). However, the absolute and relative sex difference in ASRs showed a slight declining trend during 1990 and 2019, with absolute difference decreasing from 1818.23 to 1602.58 (Fig. 1b), and relative difference decreasing from 1.71 to 1.61 (Fig. 1c).

The global and WHO regional incident cases and ASRs of MDD, for males and females, in 1990 and 2019, were shown in Table 1. Worldwide, females had a higher risk of MDD than males in 1990 (RR: 1.706; 95% CI: 1.705–1.706) and 2019 (RR: 1.602; 95% CI: 1.619–1.620). The highest RRs were observed in the Region of the Americas, being 1.925 (95% CI: 1.924–1.927) in 1990 and 1.929 (95% CI: 1.928–1.930) in 2019.

**Table 1:** The global and WHO regional incidence of MDD for both sexes in 1990 and 2019

Year and region	Incident cases (95% confidence interval) (thousands)		Age-standardized incidence rates (95% confidence interval) (per 100,000)		Risk ratio (95% confidence interval)
	Male	Female	Male	Female	
*1990*					
Global	63,182.2 (54,925.0 to 72,297.6)	109,538.7 (95,721.3 to 125,047.5)	2578.8 (2266.1 to 2925.5)	4397.1 (3869.1 to 4976.1)	1.705 (1.705 to 1.706)
African Region	7146.9 (6115.0 to 8306.0)	11,130.5 (9443.1 to 13,035.6)	4073.5 (3559.1 to 4659.9)	6060.9 (5251.7 to 6944.4)	1.488 (1.487 to 1.489)
Eastern Mediterranean Region	5519.0 (4664.0 to 6500.7)	8742.9 (7341.4 to 10,316.3)	3648.3 (3140.3 to 4242.9)	6122.6 (5217.5 to 7149.3)	1.678 (1.676 to 1.680)
European Region	12,282.4 (10,877.3 to 13,821.6)	23,931.9 (21,291.7 to 26,713.2)	2798.5 (2481.9 to 3145.4)	4851.1 (4309.5 to 5442.4)	1.733 (1.732 to 1.735)
Region of the Americas	8344.4 (7287.9 to 9589.8)	17,077.5 (14,872.2 to 19,425.9)	2474.4 (2179.5 to 2825.0)	4764.2 (4156.0 to 5403.8)	1.925 (1.924 to 1.927)
South-East Asia Region	16,350.1 (13,972.2 to 18,953.9)	24,101.6 (20,754.8 to 27,997.3)	2997.3 (2602.4 to 3434.2)	4571.5 (3969.2 to 5272.3)	1.525 (1.524 to 1.526)
Western Pacific Region	13,329.1 (11,461.5 to 15,323.4)	24,236.8 (20,894.0 to 27,740.2)	1696.9 (1485.0 to 1922.2)	3129.9 (2739.0 to 3551.3)	1.845 (1.843 to 1.846)
*2019*					
Global	103,718.7 (90,683.7 to 118,044.6)	171,085.1 (150,207.7 to 195,228.6)	2672.5 (2336.6 to 3041.6)	4436.3 (3894.9 to 5062.3)	1.660 (1.660 to 1.660)
African Region	15,488.9 (13,186.0 to 18,080.8)	24,011.6 (20,414.6 to 28,011.1)	3918.7 (3418.7 to 4483.0)	5635.2 (4885.9 to 6487.1)	1.438 (1.437 to 1.439)
Eastern Mediterranean Region	12,612.9 (10,705.5 to 14,833.4)	19,182.3 (16,125.4 to 22,510.3)	3634.6 (3132.5 to 4236.6)	6028.2 (5162.7 to 7022.0)	1.659 (1.657 to 1.660)
European Region	13,806.2 (12,117.1 to 15,637.1)	26,275.5 (23,160.4 to 29,779.1)	2640.4 (2309.8 to 3004.1)	4627.4 (4038.9 to 5298.4)	1.753 (1.751 to 1.754)
Region of the Americas	14,353.7 (12,638.6 to 16,274.4)	28,817.7 (25,427.0 to 32,383.4)	2716.1 (2386.1 to 3085.4)	5238.6 (4607.5 to 5909.0)	1.929 (1.928 to 1.930)
South-East Asia Region	28,512.4 (24,809.3 to 32,555.8)	40,607.4 (35,300.3 to 46,524.7)	2832.9 (2476.4 to 3212.3)	4018.4 (3497.7 to 4600.1)	1.419 (1.418 to 1.419)
Western Pacific Region	18,554.8 (16,309.0 to 20,938.5)	31,617.6 (27,923.0 to 35,522.7)	1614.5 (1417.7 to 1823.9)	2711.2 (2388.2 to 3064.8)	1.679 (1.678 to 1.680)

WHO, World Health Organization; MDD, major depressive disorder

### Sex difference in incidence by age

**Fig. 2:**
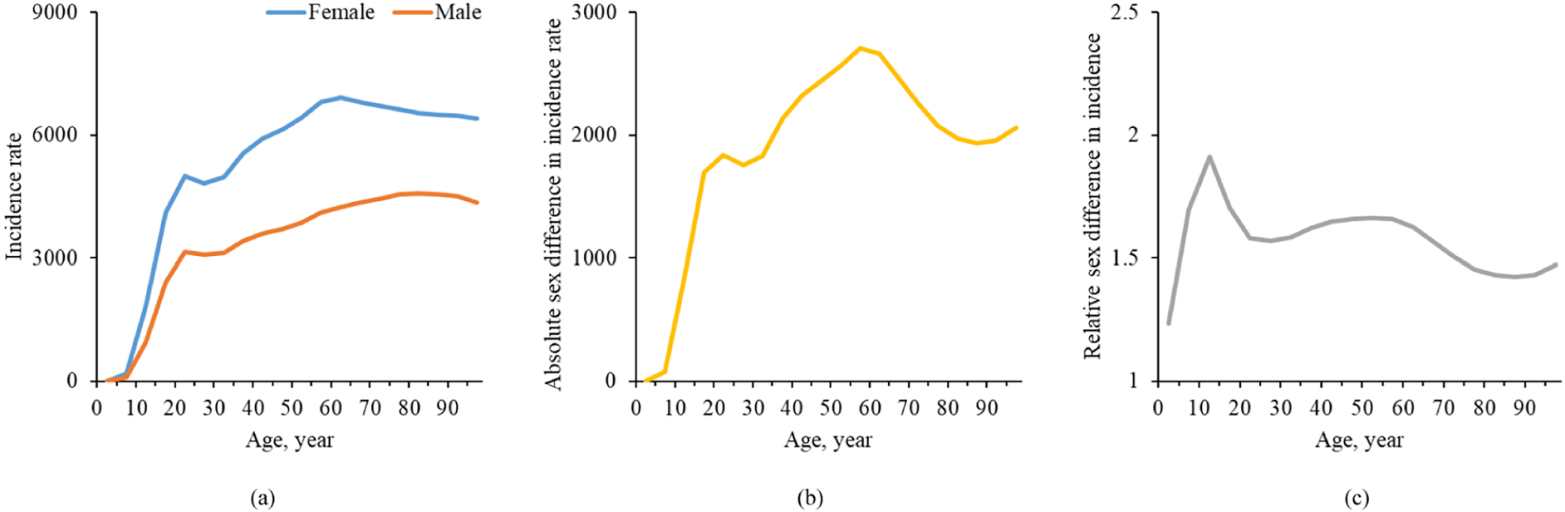
Sex difference in global incidence of major depressive disorder in different age groups in 2019, in terms of **a** incidence rates, **b** absolute (female minus male) sex difference in incidence rates, and **c** relative (female to male ratio) sex difference in incidence rates

In 2019, global incidence rates in both sexes increased rapidly with age for those under 20 years old. The highest incidence rates were 4576.43 (95% UI: 3309.49–5877.25) in the age group of 80–84 for males, and 6914.76 (95% UI: 5311.00–8649.03) in the age group of 60–64 for females (Fig. 2a). The absolute sex difference in incidence rates increased rapidly with age for those under 20 years old, with the greatest absolute difference being 2705.03 in the age group of 55–59 (Fig. 2b). The relative sex difference in incidence rates increased rapidly with age for those under 15 years old, with the greatest relative difference being 1.91 in the age group of 10–14 (Fig. 2c). The global age-specific incident cases and incidence rates of MDD, for males and females, in 2019, were shown in Table 2. The highest RR (RR:1.913; 95% CI: 1.910–1.915) was observed in the age group of 10–14.

**Table 2:** The global age-specific incidence of MDD for both sexes in 2019

Age (years)	Incident cases (95% confidence interval) (thousands)		Incidence rates (95% confidence interval) (per 100,000)		Risk ratio (95% confidence interval)
	Male	Female	Male	Female	
All age	103,718.7 (90,683.7 to 118,044.6)	171,085.1 (150,207.7 to 195,228.6)	2672.5 (2336.6 to 3041.6)	4436.3 (3894.9 to 5062.3)	1.660 (1.660 to 1.660)
< 5	5.7 (2.1 to 13.7)	6.5 (2.5 to 15.6)	1.7 (0.6 to 4.0)	2.0 (0.8 to 4.9)	1.236 (1.193 to 1.281)
5–9	363.7 (167.5 to 652.5)	578.3 (281.3 to 996.3)	107.6 (49.6 to 193.1)	182.6 (88.8 to 314.5)	1.696 (1.689 to 1.703)
10–14	3151.0 (1775.0 to 4940.0)	5654.4 (3332.6 to 8764.1)	951.0 (535.7 to 1490.9)	1819.0 (1072.1 to 2819.4)	1.913 (1.910 to 1.915)
15–19	7670.5 (5468.0 to 10,599.0)	12,412.3 (8857.0 to 17,048.1)	2413.8 (1720.7 to 3335.3)	4113.3 (2935.1 to 5649.6)	1.704 (1.703 to 1.706)
20–24	9608.7 (6737.4 to 12,958.4)	14,771.4 (10,235.2 to 19,851.8)	3156.9 (2213.6 to 4257.5)	4994.1 (3460.5 to 6711.8)	1.582 (1.581 to 1.583)
25–29	9378.2 (6690.7 to 13,057.8)	14,536.5 (10,075.4 to 20,201.2)	3077.1 (2195.3 to 4284.4)	4834.3 (3350.7 to 6718.2)	1.571 (1.570 to 1.572)
30–34	9510.1 (6924.6 to 13,025.0)	14,840.3 (10,767.3 to 20,408.6)	3136.9 (2284.0 to 4296.2)	4970.7 (3606.4 to 6835.7)	1.585 (1.583 to 1.586)
35–39	9335.2 (6962.1 to 11,988.3)	14,922.2 (10,990.4 to 19,213.8)	3425.3 (2554.5 to 4398.7)	5559.0 (4094.2 to 7157.7)	1.623 (1.622 to 1.624)
40–44	8941.8 (6501.5 to 11,449.3)	14,481.6 (10,552.2 to 18,632.4)	3594.8 (2613.7 to 4602.8)	5918.1 (4312.3 to 7614.4)	1.646 (1.645 to 1.648)
45–49	8824.9 (6920.3 to 10,811.4)	14,463.8 (11,412.3 to 17,670.0)	3702.1 (2903.1 to 4535.5)	6143.6 (4847.5 to 7505.5)	1.659 (1.658 to 1.661)
50–54	8404.8 (6728.4 to 10,316.1)	14,094.7 (11,362.2 to 17,207.6)	3862.7 (3092.3 to 4741.2)	6429.1 (5182.7 to 7849.1)	1.664 (1.663 to 1.666)
55–59	7495.0 (5744.7 to 9565.3)	12,812.3 (9790.2 to 16,163.7)	4101.0 (3143.2 to 5233.7)	6806.0 (5200.6 to 8586.3)	1.660 (1.658 to 1.661)
60–64	6472.9 (4992.6 to 8168.7)	11,089.9 (8517.8 to 13,871.4)	4254.2 (3281.3 to 5368.7)	6914.8 (5311.0 to 8649.0)	1.625 (1.624 to 1.627)
65–69	5376.5 (4440.3 to 6448.4)	9188.2 (7592.0 to 10,965.2)	4349.4 (3592.0 to 5216.4)	6807.7 (5625.1 to 8124.4)	1.565 (1.564 to 1.567)
70–74	3925.4 (3171.1 to 4810.2)	6645.1 (5425.4 to 8071.4)	4455.4 (3599.2 to 5459.7)	6713.4 (5481.2 to 8154.3)	1.507 (1.505 to 1.509)
75–79	2604.4 (1880.8 to 3381.4)	4628.0 (3367.1 to 5999.8)	4552.7 (3287.9 to 5911.1)	6625.7 (4820.6 to 8589.8)	1.455 (1.453 to 1.457)
80–84	1612.6 (1166.2 to 2071.0)	3222.3 (2321.2 to 4110.5)	4576.4 (3309.5 to 5877.2)	6551.5 (4719.3 to 8357.2)	1.432 (1.429 to 1.434)
85–89	741.8 (574.1 to 931.9)	1764.8 (1368.2 to 2193.8)	4555.4 (3525.7 to 5723.1)	6489.0 (5030.8 to 8066.3)	1.424 (1.421 to 1.428)
90–94	239.7 (170.9 to 327.3)	747.9 (535.9 to 1010.7)	4515.7 (3220.3 to 6166.3)	6475.7 (4639.7 to 8750.6)	1.434 (1.428 to 1.440)
95 plus	55.6 (33.3 to 83.2)	224.4 (137.7 to 333.3)	4356.9 (2609.9 to 6520.3)	6418.5 (3938.9 to 9532.8)	1.473 (1.460 to 1.487)

MDD, major depressive disorder

### Sex difference in incidence by socioeconomic development

**Fig. 3:**
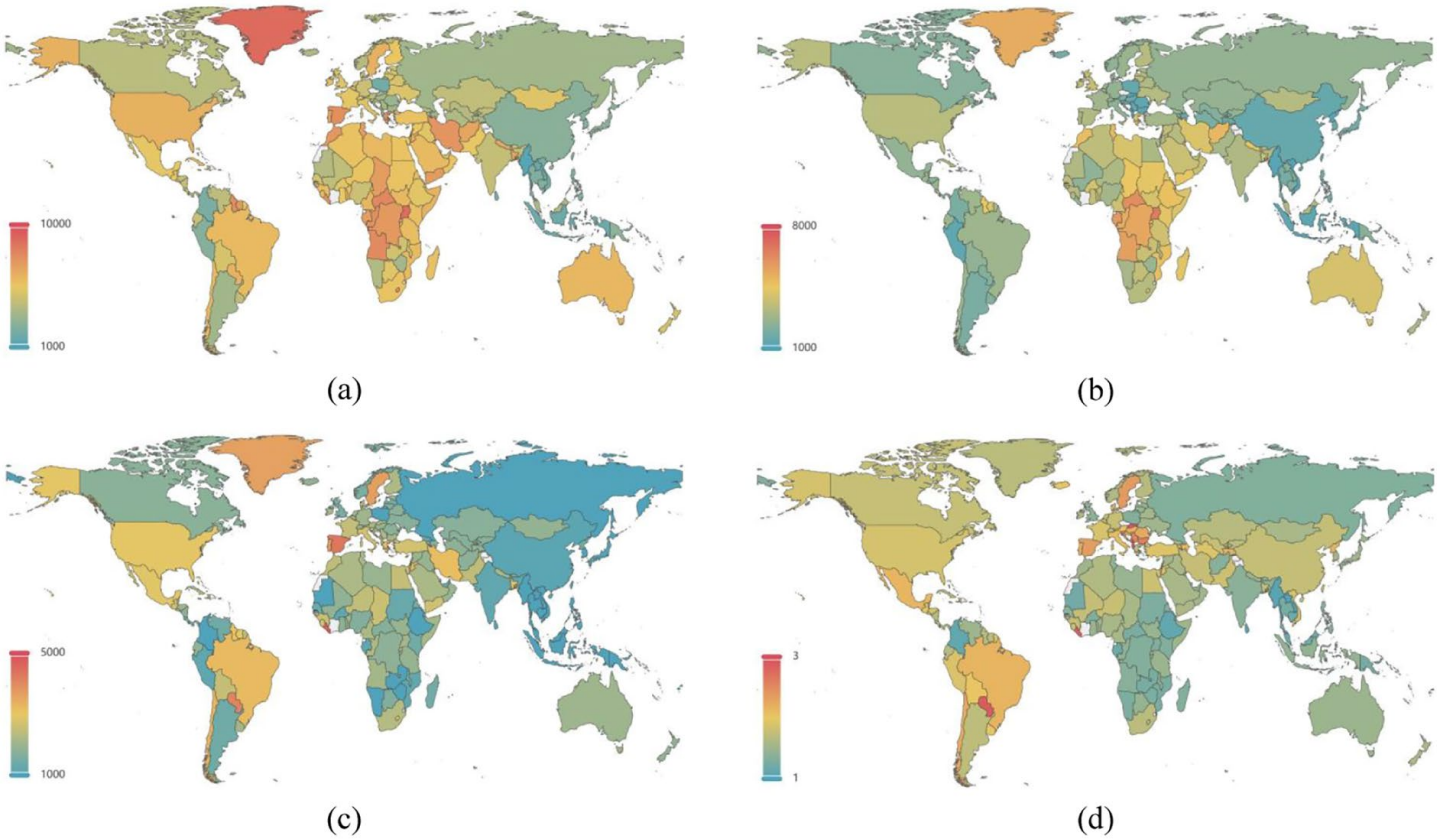
Sex difference in national incidence of major depressive disorder in 2019, in terms of **a** age standardized incidence rates in females, **b** age standardized incidence rates in males, **c** absolute (female minus male) sex difference in age standardized incidence rates, and **d** relative (female to male ratio) sex difference in age standardized incidence rates

**Fig. 4:**
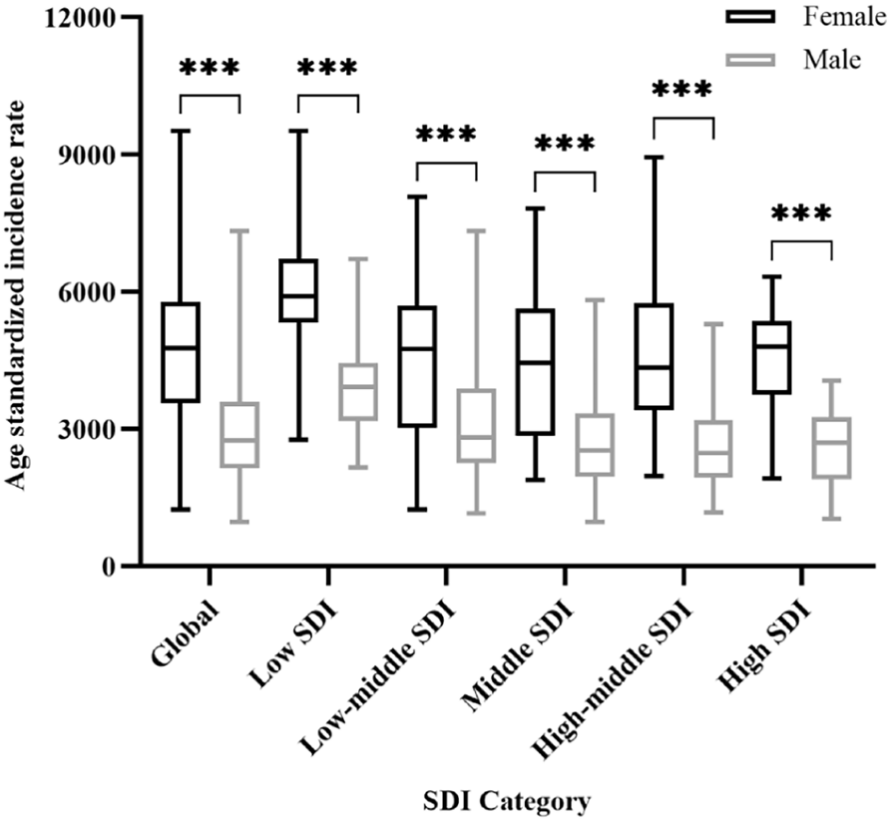
Age-standardized incidence rates among females were higher than that among males for SDI-based country groups. Lines inside the boxes indicate the medians, boxes the 25th and 75th percentiles, and lines outside the boxes the minimum and the maximum. *** indicates *P* < .001. SDI, socioeconomic development index

ASRs for males and females, 204 countries and territories, in 2019, were demonstrated in Fig. 3a and b. Mann–Whitney U test showed that females had significant higher ASRs of MDD than males for 204 countries and territories (Z = − 11.15, P < 0.001), with median (interquartile range) of ASRs being 2746.65 (2136.81–3583.68) for males and 4774.96 (3548.68–5791.83) for females. Multiple comparisons revealed that ASRs for females were significantly higher than that for males, for low SDI group (males vs. females: 3915.58 (3173.72–4449.27) vs. 5901.45 (5334.44–6712.99); Z = − 5.50, P < 0.001), low-middle SDI group (2819.09 (2258.60–3870.29) vs. 4749.86 (3012.33–5695.73); Z = − 4.13, P < 0.001), middle SDI group (2523.49 (1952.90–3328.19) vs. 4438.89 (2846.17–5628.38); Z = − 4.61, P < 0.001), high-middle SDI group (2473.85 (1938.66–3185.60) vs. 4345.08 (3405.54–5751.71); Z = − 6.07, P < 0.001), and high SDI group (2690.36 (1899.22–3254.34) vs. 4810.10 (3741.17–5368.61); Z = − 6.19, P < 0.001) (Fig. 4).

**Fig. 5:**
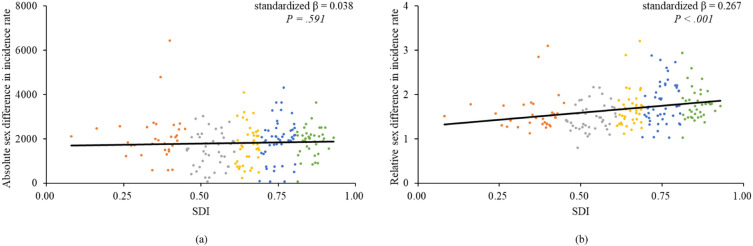
Sex difference in incidence of major depressive disorder by SDI. Linear relationship **a** between absolute (female minus male) sex difference in age standardized incidence rates and SDI, and **b** between relative (female to male ratio) sex difference in age standardized incidence rates and SDI. SDI, socioeconomic development index

Absolute and relative sex difference in ASRs for males and females, 204 countries and territories, in 2019, were demonstrated in Fig. 3C and D. Despite that absolute sex difference in ASRs was not related to SDI, relative sex difference had a significant positive relationship with SDI (standardized β = 0.267, P < 0.001) (Fig. 5).

## Discussion

Sex difference in global MDD incidence showed a slight declining trend from 1990 to 2019. However, females always have a higher incidence than males over the past decades. Notably, sex difference in incidence rates increased rapidly with age for young people, with the highest RR observed in the age group of 10–14. Last but not least, countries with higher levels of socioeconomic development were found to have greater sex difference in MDD incidence.

Globally, the incidence of MDD in both sexes did not change so much in the past three decades. Females have a higher incidence of MDD than males in all WHO regions, especially the Region of the Americas (females have about twice the risk as males). Four gender-related subtypes of MDD have been proposed, namely early onset, developmental, reproductive, and pathophysiological subtypes, of which the developmental subtype has the greatest potential to contribute to the gender gap [8]. Biological factors (such as genetic risk, hormones, and physiological stress response), psychological factors (such as temperament, personality and coping styles) and environmental factors (societal structural gender inequality) are potentially responsible for the gender gap in MDD [8]. Cross-national analyses revealed that smaller gender gap in MDD were found in countries with greater gender equality [17]. Evidence of decreasing gender gap has been found in countries in which gender roles of females have improved, in terms of opportunities for employment, educational achievement, and other indicators of increasing gender equality [18]. Not surprisingly, trend studies in countries in which gender roles have been static failed to document a reduction in gender gap in MDD [19, 20].

The incidence of MDD is commonly low before puberty, whereas MDD increases much more substantially in females, to about twice that of males, during puberty [11, 21, 22]. Both early pubertal timing and advancing pubertal stage are associated with the onset of females’ increasing risk of MDD [22, 23]. In contrast with males, early pubertal timing in females is linked to more severe and longer-lasting psychopathology such as depressive disorders [23]. Contextual adversities, such as childhood sexual abuse and poor parent–child relationship, predict early puberty in females, but not in males. Thus, female pubertal transition tends to be more sensitive to adverse environments than the male transition [23]. In addition, evidence of the interactions of activating sex hormones, intrapersonal susceptibility, and interpersonal factors has been provided to explain the extraordinarily high increase in depression rates during pubertal transition in females [24, 25]. Against popular belief, sex difference in MDD does not become smaller after puberty. From early adulthood to late in life, the incidence of MDD in both sexes followed a parallel course, with persistently predominant incidence in females [11]. Multiple variables, such as stressors, coping styles, interpersonal orientation, and social support, might predict the sex difference in MDD throughout the life span [26]. Clarifying the interactions between the predictors of sex difference and the lifespan development could promote timely diagnosis and treatment of MDD throughout the full life cycle.

For each SDI-based country group, the incidence of MDD were higher in females than that in males. Greater relative sex difference was found in countries with higher levels of socioeconomic development. By analyzing disability adjusted of life years caused by depressive disorders from the GBD 2015, a positive relationship between log-transformed female to male ratio of depression rates and national gross domestic product had been found, after adjusting for regional effects and other socioeconomic factors, which was consistent with our findings [27].﻿ In less developed countries, female roles are narrowly prescribed, and choice of roles and conflict between different roles is minimized. In more developed countries, females' lives are less predetermined and they have a wider range of choices of careers and life styles. Greater choice may mean more conflict between possible roles (such as the mother, partner, work and homemaker role) [28]. High work-family conflicts have been proposed to be related to the development of depressive symptoms in females [29]. A representative European survey revealed that females living alone and particularly employed females had higher depression scores [17]. Traditional female roles encourage cooperation and dependency. However, modern societies encourage individual achievement, self-reliance and independence for females especially in developed countries [30]. Thus, help seeking for depression carried greater psychological costs because help seeking violates modern female roles. On one hand, stigma about depression was found to be related to less help seeking among patients with MDD [31]. On the other hand, females who are more depressed and who have higher levels of psychological distress experience their condition as more stigmatizing [32]. There exists a vicious cycle of depression, stigma and help seeking behavior. Therefore, public awareness and understanding of depression should be promoted in society, hopefully leading to reduced stigma and increased help-seeking, especially for modern females. Another reason why higher sex difference in MDD incidence was found in more developed countries may also be linked to better estimates from more developed countries which allowed a better differentiation between gender, while estimates from less developed countries may be less precise.

This study was subject to the limitations of the GBD 2019, for example, data sources and statistical methods, as reported in the GBD 2019 [11]. ﻿The major limitation of the GBD analysis was the availability of primary data. Where data are not available, the results depend on the out-of-sample predictive validity of the modelling efforts. ﻿While improvements to data processing and modelling can lead to incremental improvements in the accuracy of GBD estimates, fundamental improvements require more and better primary data collection. Even when data are available, they might not have been obtained using the preferred case definition or measurement method. Due to the use of aggregate data at the national level instead of district data, potential bias may be introduced by geographic variations in incidence estimates. Although this study presented a global view of sex difference in MDD incidence, the conclusions may not hold up to a specific district. Considering that updates of GBD data will be available in the future, sex difference in global MDD incidence during a longer period of time could be further explored. Results from emerging studies indicated that COVID-19 pandemic affected male and female populations in different ways [33]. Females seem to experience less severe short-term complications but suffer worse long-term COVID complications, including depression, reduced physical activity, and deteriorating lifestyle habits.

## Conclusions

In conclusion, this study revealed that although slight improvement in sex difference in global MDD incidence has been achieved, sex difference still persists in the past decades, with females always having a higher incidence than males. Sex difference increased rapidly with age for young people and was greater in more developed countries. Despite that MDD is treatable with therapy and medications, sex difference in MDD remains a major global public health problem. Findings from this study call attention on the female vulnerability to MDD, and might provide important clues for making sex-specific health policy to reduce sex difference in MDD. ﻿A coordinated response by governments and the global health community is urgently needed to address the present and future gender gap in mental health.

## Data Availability

The datasets analysed during the current study are available from the publicly available website: http://ghdx.healthdata.org/gbd-results-tool.

## Abbreviations

ASRs: Age-standardized incidence rates
GBD: Global Burden of Disease Study
MDD: Major depressive disorder
RR: Risk ratios
SDI: Socioeconomic development index
WHO: World Health Organization
